# Environment-Specific Probiotic Supernatants Modify the Metabolic Activity and Survival of *Streptococcus mutans in vitro*

**DOI:** 10.3389/fmicb.2020.01447

**Published:** 2020-06-26

**Authors:** Haiyue Yu, Petra Ganas, Falk Schwendicke

**Affiliations:** Charité–Universitätsmedizin Berlin, corporate member of Freie Universität Berlin, Humboldt-Universität zu Berlin, and Berlin Institute of Health, Department of Oral Diagnosis, Digital Health and Health Services Research, Berlin, Germany

**Keywords:** caries, colony forming units, dental, lactate production, metabolites, probiotics

## Abstract

A range of studies showed probiotics like *Streptococcus oligofermentans* and *Limosilactobacillus reuteri* to inhibit the cariogenic activity and survival of *Streptococcus mutans*, possibly via the production of substances like H_2_O_2_, reuterin, ammonia and organic acids. We aimed to assess the environment-specific mechanisms underlying this inhibition. We cultured *L. reuteri* and *S. oligofermentans* in various environments; minimal medium (MM), MM containing glucose (MM+Glu), glycerol (MM+Gly), lactic acid (MM+Lac), arginine (MM+Arg) and all four substances (MM+all) *in vitro*. Culture supernatants were obtained and metabolite concentrations (reuterin, ammonia, H_2_O_2_, lactate) measured. *S. mutans* was similarly cultivated in the above six different MM variation media, with glucose being additionally added to the MM+Gly, MM+Lac, and MM+Arg group, with (test groups) and without (control groups) the addition of the supernatants of the described probiotic cultures. Lactate production by *S. mutans* was measured and its survival (as colony-forming-units/mL) assessed. *L. reuteri* environment-specifically produced reuterin, H_2_O_2_, ammonia and lactate, as did *S. oligofermentans*. When cultured in *S. oligofermentans* supernatants, lactate production by *S. mutans* was significantly reduced (*p* < 0.01), especially in MM+Lac+Glu and MM+all, with no detectable lactate production at all (controls means ± SD: 4.46 ± 0.41 mM and 6.00 ± 0.29 mM, respectively, *p* < 0.001). A similar reduction in lactate production was found when *S. mutans* was cultured in *L. reuteri* supernatants (*p* < 0.05) for all groups except MM+Lac+Glu. Survival of *S. mutans* cultured in *S. oligofermentans* supernatants in MM+Lac+Glu and MM+all was significantly reduced by 0.6-log_10_ and 0.5-log_10_, respectively. Treatment with the supernatant of *L. reuteri* resulted in a reduction in the viability of *S. mutans* in MM+Gly+Glu and MM+all by 6.1-log_10_ and 7.1-log_10_, respectively. Probiotic effects on the metabolic activity and survival of *S. mutans* were environment-specific through different pathways.

## Introduction

The human oral cavity harbors more than 700 microbial species, which constitute a dynamic microbial community (Aas et al., 2005). The coexistence and competition between different species are central to the oral microbial homeostasis (Bao et al., 2015). A disturbance in this homeostasis, termed dysbiosis, is associated with dental diseases like dental caries or periodontitis (Exterkate et al., 2010). For dental caries, the dominance of acidogenic and aciduric species like *Streptococcus mutans*, triggered by the abundant intake of fermentable carbohydrates, is associated with a net mineral loss from dental hard tissues and the formation of a caries lesion (Marsh, 2006).

Contemporary caries management aims to rebuild a healthy microbial equilibrium within the dental biofilm (Marsh, 2006; Bao et al., 2015). One strategy supposedly supporting such rebalancing of the biofilm is the application of probiotics. Probiotics are microorganisms, mainly bacteria, that when administered in sufficient amounts, provide health benefits to the host (Ng et al., 2009; Bosch et al., 2012), for example by inhibiting the metabolic activity and survival of harmful microbiota as well as modulating the host’s immune response, thereby helping to stabilize the local microecosystem (Meurman, 2005). Probiotics have been tested both *in vitro* and in clinical studies for their anti-caries effect, with mixed results (Jalasvuori et al., 2012; Gruner et al., 2016).

Certain probiotics have been tested more widely. *Streptococcus oligofermentans*, a synonym of *Streptococcus cristatus* (Jensen et al., 2016) was isolated from healthy tooth surfaces (Tong, 2003) and has anti-bacterial effects against pathogens like *S. mutans* (Liu et al., 2014). It produces hydrogen peroxide (H_2_O_2_) from lactic acid (Tong et al., 2007, 2008) as well as ammonia from arginine, which may both reduce the amount of free lactic acid, thereby increasing the local pH and preventing the initiation of a caries lesion or slowing down or stopping lesion progression (Burne and Marquis, 2000; Clancy et al., 2000). *Limosilactobacillus reuteri* (Zheng et al., 2020) is an obligate heterofermentative probiotic and most strains in its human lineages have the ability to excrete reuterin (Mu et al., 2018), a potent antibiotic substance, which exhibits broad-spectrum antimicrobial effect on Gram-positive and Gram-negative bacteria (Talarico and Dobrogosz, 1989; Doleyres et al., 2005). In addition to reuterin, *L. reuteri* also produces H_2_O_2_, organic acid (Kang et al., 2011) and ammonia (Mu et al., 2018; Zaura and Twetman, 2019), with possible impact on *S. mutans* metabolic activity and survival. Furthermore, some strains of *L. reuteri* generate a unique antagonistic activity, reutericyclin, which shows a broad inhibitory spectrum but has no effect on the growth of gram-negative bacteria (Ganzle et al., 2000; Lin et al., 2015).

The antibacterial effect of these probiotics hence relies, at least in parts, on the production of the described substances. This production, in turn, is likely to be dependent on the environmental conditions, especially the availability of certain educts required to produce reuterin, H_2_O_2_ etc., So far, it was not studied if different environments modify the probiotic effects on cariogenic pathogens like *S. mutans*. Deeper knowledge on such environmental requirements is needed both for future research (setting up appropriate models considering these requirements) and for clinical applications. For example, it may be feasible to boost the probiotic anti-caries effect by supplementing probiotic products with certain substances required for a specific probiotic activity.

We aimed to assess the environment-specific activity and impact of two different probiotics, *S. oligofermentans*, and *L. reuteri*, on the metabolic activity and survival of *S. mutans*. We hypothesized that the metabolic activity and survival of *S. mutans* is significantly reduced when exposed to probiotic supernatants, and that this effect is environment-specific.

## Materials and Methods

This study used an established *in vitro* model (Ganas and Schwendicke, 2019) to assess the environment-specific impact of probiotics on metabolic activity and survival of *S. mutans*. Different environmental conditions were simulated by using determined modifications of a saliva analog, allowing to deterministically vary the metabolic activity of the two different probiotics, *S. oligofermentans*, and *L. reuteri.* The supernatants resulting from the cultivation of probiotics in different environments were then used to assess their impact on *S. mutans* metabolic activity, measured via determining the lactate production, and survival, measured via the colony-forming-units/mL of *S. mutans.* Controls of *S. mutans* cultured in different environments, but without probiotic supernatant, were additionally used. All assays and tests were performed in three biological replications, each with two technical replications (measurements) whose average was used for statistical analysis.

### Bacterial Strains and Growth Conditions

Three bacterial strains *S. mutans*, DSM 20523, *S. oligofermentans*, DSM 8249 (DMSZ, Braunschweig, Germany) and *L. reuteri*, ATCC PTA 5289 (BioGaia, Stockholm, Sweden) were used. The strains *S. mutans* and *S. oligofermentans* were grown on blood agar plates COLS+ (Oxoid, Wesel, Germany) while the strain *L. reuteri* was maintained on deMan-Rogosa-Sharpe (MRS) agar (Oxoid) at 37°C aerobically for 1–2 days.

### Preparation of Deproteinized Supernatants From Probiotics Cultures

The two probiotics were precultured separately in brain-heart-infusion (BHI) broth (Carl Roth, Karlsruhe, Germany) supplemented with 1% glucose (Carl Roth), 4 g/L yeast extract and 8 g/L beef extract (Carl Roth) for 18 h under aerobic conditions at 37°C in 15 ml Falcon tubes (Corning, Kaiserslautern, Germany). After centrifugation at 7100 *g* for 15 min at room temperature, the supernatants were removed and the cells were transferred to 0.9% sodium chloride with an inoculum (means ± SD) of 3.96 ± 0.37 × 10^7^ cells/mL for *S. oligofermentans* and 4.16 ± 0.22 × 10^7^ cells/mL for *L. reuteri*, with a total of 6 ml-cultures in 15 ml Falcon tubes (Corning), respectively. After another centrifugation at 7100 *g* for 15 min, the supernatants were discarded and 6 mL Minimal Medium (MM) was added.

The minimal medium was based on a chemically defined saliva analog (Wong and Sissions, 2001) and consisted of 10 mM KH_2_PO4 (Merck, Darmstadt, Germany), 10 mM K_2_HPO4 (Carl Roth), 1 mM NaCl (Carl Roth), 3 mM KCl (Merck), 0.2 mM NH_4_Cl (Merck), and 0.2 mM MgCl_2_ × 6H_2_O (Merck). The MM was supplemented as follows to generate six different MM variations, allowing to assess the relevance of the metabolic environment on the probiotic activity and its association with the inhibition of *S. mutans* survival and activity: (1) MM, (2) MM with 5 mM glucose (Carl Roth) (MM+Glu), (3) MM with 300 mM glycerol (Sigma-Aldrich, Taufkirchen, Germany) (MM+Gly), (4) MM with 5 mM Lactic acid (Carl Roth) (MM+Lac), (5) MM with 5 mM arginine (Sigma-Aldrich) (MM+Arg) and (6) MM with 5 mM glucose, 300 mM glycerol, 5 mM Lactic acid and 5 mM arginine (MM+all). The final pH of the six MM variations was MM 6.95, MM+Glu 6.90, MM+Gly 6.92, MM+Lac 6.61, MM+Arg 7.14, MM+all 6.83. For the culture of *S. mutans*, in order to enable it to produce lactate, glucose was added to MM+Gly, MM+Lac, MM+Arg group to a final concentration of 5 mM.

In a pre-experiment, the cultivation period of the probiotics was varied (15 min, 30 min, 2, 4, and 18 h) to gauge the impact of this period on the production of H_2_O_2_, lactate, reuterin and ammonia. Different probiotic cultivation periods were eventually used to generate supernatants optimally enriched with these metabolites. As a consequence, *S. oligofermentans*, was cultured in MM+Glu and MM+Lac aerobically at 37°C for 30 min, in MM and MM+Gly for 2 h, and in MM+Arg and MM+all for 4 h, followed by centrifugation at 20800 *g* for 10 min at room temperature to obtain the supernatants. *L. reuteri* was cultured in MM+Glu, MM+Gly, MM+Lac and MM+all for 4 h and in MM and MM+Arg for 18 h, followed by the same protocol to collect supernatants. Deproteinization was conducted using Amicon Ultra-2ml centrifugal filter units with molecular weight cut-off (MWCO) of 10 kDa (Merck) at 7500 *g* for 20 min at room temperature. The deproteinized supernatants were maintained at −80°C for later processing. Control samples without bacteria were established using BHI medium containing 1% Glucose, 4 g/L yeast extract and 8 g/L beef extract followed by six different MM variations treated in the same way as in the bacterial culture groups.

### Preparation of Deproteinized Supernatants From *S. mutans* Cultures

*S. mutans* was precultured in the BHI+1% glucose+4 g/L yeast extract+8 g/L beef extract medium for 18 h aerobically at 37°C in 15 mL Falcon tubes. After centrifugation at 7100 *g* for 15 min at room temperature, cultivation supernatants were removed. Cells were rinsed with 0.9% sodium chloride and after another centrifugation at 7100 *g* for 15 min, bacteria were transferred to MM, MM+Glu, MM+Gly+Glu, MM+Lac+Glu, MM+Arg+Glu and MM+all media, inoculated with 4.35 ± 0.39 × 10^7^ cells as 1 mL-cultures in 1.5 mL Eppendorf tubes (Eppendorf, Hamburg, Germany), followed by incubation at 37°C aerobically for further 18 h. Control samples without bacteria were established using BHI medium containing 1% Glucose, 4 g/L yeast extract and 8 g/L beef extract followed by six different MM variations treated in the same way as in the bacterial culture groups, except that 5 mM glucose was additionally added to MM+Gly, MM+Lac, MM+Arg groups. Afterward, the deproteinized supernatants were collected and stored as described above.

### Culture of *S. mutans* With Probiotic Supernatants

After pre-incubation in BHI+1% glucose+4 g/L yeast extract+8 g/L beef extract medium for 18 h, *S. mutans* was treated in the same manner as above with an inoculum of 4.29 ± 0.65 × 10^7^ cells/mL. After centrifugation, the supernatants were discarded and 1 mL of the supernatants of *S. oligofermentans* and *L. reuteri* (cultured in the different MM as described) were pipetted into 1.5 mL Eppendorf tubes (Eppendorf). Glucose was additionally added to the MM+Gly, MM+Lac, and MM+Arg at a final concentration of 5 mM. *S. mutans* was cultured for further 18 h as before. The probiotic supernatant without bacteria was used as control. The deproteinized supernatants of *S. mutans* were collected and stored as described above.

### Metabolite Assays

The metabolite production of lactate, H_2_O_2_ and ammonia was measured via assessing their concentration in the deproteinized supernatants using colorimetric assay kits (Sigma-Aldrich) in accordance with the manufacturers’ instructions. The determination of reuterin in the supernatants was analyzed as described elsewhere (Kang et al., 2011) with some modifications. In short, 250 μL of deproteinized probiotic supernatant samples were added to 187.5 μL of 10 mM tryptophan dissolved in 0.05 M HCl, followed by 750 μL of 37% HCl. Under acidic conditions, tryptophan and the aldehyde of reuterin form a β-carboline derivative which oxidizes to produce a purple pigment. After incubation at 37°C for 20 min, the absorbance was measured at 560 nm. Acrolein (Sigma) was used as the calibration standard. To obtain standard curve, 0–15 μmol of acrolein was added to 1 mL of distilled water. The detection of absorbance was performed by the 96 well plate spectrophotometer Multiskan Go (Thermo Fisher Scientific, Schwerte, Germany).

### Viable Bacteria Enumeration

Viable bacteria cells were determined by plating 100 μL aliquots of 1 mL serial dilutions on COLS+ agar plates for *S. oligofermentans* and *S. mutans* or on MRS agar plates for *L. reuteri.* After 1–2 days of aerobic incubation at 37°C, the colony forming units/mL (CFU/mL) were calculated.

### Statistical Analysis

Descriptive analysis was performed, and one-way ANOVA followed by Dunnett’s test conducted, with *P* < 0.05 considered as statistically significant. SPSS Version 20.0 software (SPSS Inc., Chicago, IL, United States) was used for statistical analysis.

## Results

### Concentration of Metabolites of *S. oligofermentans* Under Different Environmental Conditions

**FIGURE 1:**
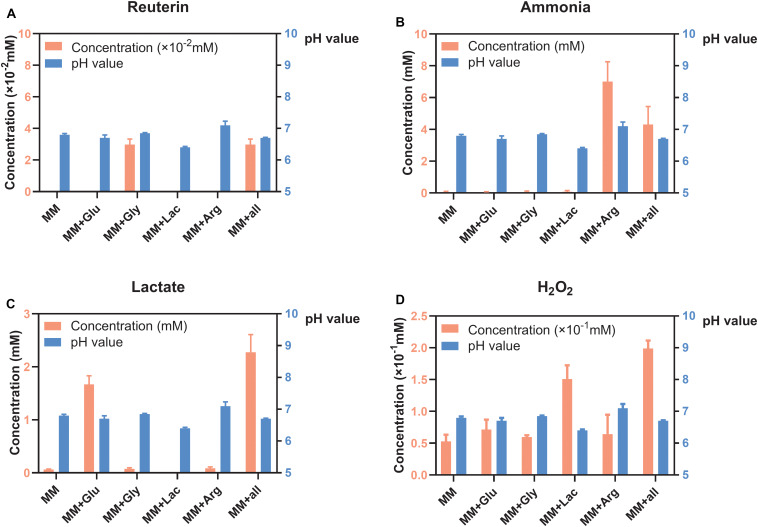
Concentration of metabolites and pH of *S. oligofermentans* in six different MM media (means ± standard deviations, *n* = 3); minimal medium (MM), MM with glucose (MM+Glu), MM with glycerol (MM+Gly), MM with Lactic acid (MM+Lac), MM with arginine (MM+Arg) and all-full medium (MM+all). The left *Y* axis showed the concentration and the right *Y* axis showed the pH value. **(A)** Very little reuterin was produced in *S. oligofermentans*. **(B)** Ammonia was detected in MM+Arg and MM+all. **(C)** Lactate was detectable mainly in MM+Glu and MM+all. **(D)** H_2_O_2_ was produced in all groups.

*S. oligofermentans* produced very little reuterin in MM+Gly and MM+all (Figure 1A). Ammonia was detected in MM+Arg and MM+all (Figure 1B) and lactate was detectable mainly in MM+Glu and MM+all (Figure 1C). H_2_O_2_ was produced in all groups, with the highest concentration in MM+all (Figure 1D). The pH of the supernatants after the incubation of *S. oligofermentans* were MM 6.80, MM+Glu 6.70, MM+Gly 6.85, MM+Lac 6.40, MM+Arg 7.10, MM+all 6.70 (Figure 1).

### Concentration of Metabolites of *L. reuteri* in Six Different MM Medium

**FIGURE 2:**
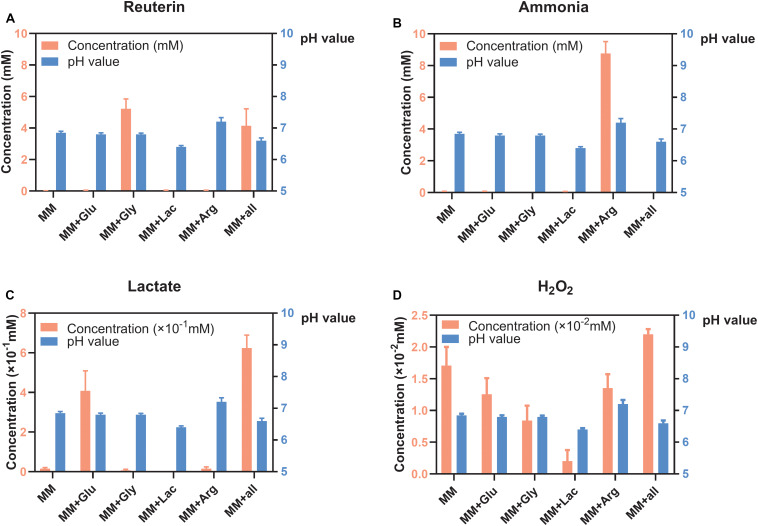
Concentration of metabolites and pH of *L. reuteri* (means ± standard deviations, *n* = 3) in six different MM media; minimal medium (MM), MM with glucose (MM+Glu), MM with glycerol (MM+Gly), MM with Lactic acid (MM+Lac), MM with arginine (MM+Arg) and all-full medium (MM+all). The left *Y* axis showed the concentration and the right *Y* axis showed the pH value. **(A)** Reuterin was detected in both MM+Gly and MM+all. **(B)** Ammonia was only detected in MM+Arg. **(C)** Lactate was mainly produced in MM+Glu and MM+all. **(D)** H_2_O_2_ was produced in all groups, the lowest being in MM+Lac.

Reuterin was detected in both MM+Gly and MM+all (Figure 2A), while ammonia was only detected in MM+Arg (Figure 2B). Lactate was detected in MM+Glu and MM+all, with only very low concentrations in the other groups (Figure 2C). H_2_O_2_ was produced in all groups, the highest concentration being measured in MM+all (Figure 2D). The pH of the supernatants after the incubation of *L. reuteri* were MM 6.85, MM+Glu 6.80, MM+Gly 6.80, MM+Lac 6.40, MM+Arg 7.20, MM+all 6.60 (Figure 2).

### Effect of Probiotic Supernatants on *S. mutans* Metabolic Activity

Lactate production of *S. mutans* was minimal (1.78 ± 0.65 × 10^–2^ mM) in control medium without glucose, and significantly higher in control media with glucose. Cultivation in supernatant of *S. oligofermentans* significantly reduced the lactate production of *S. mutans* regardless of the medium (Table 1) and, via utilization of lactate by *S. mutans*, even decreased the concentration of lactate in MM+all (concentration decreased by −2.14 ± 0.35 mM) and MM+Lac+Glu (concentration decreased by −1.39 ± 0.25 mM) respectively, (*p* < 0.001). Cultivation in supernatant of *L. reuteri* also significantly reduced the lactate production of *S. mutans* in all media except MM+Lac+Glu (Table 1).

**TABLE 1:** Changes (means ± standard deviations, *n* = 3/group) in lactate concentration (mM) of *S. mutans* (Sm) after cultivation in the supernatant (Sup.) of *S. oligofermentans* (So) and *L. reuteri* (Lr).

Metabolite (mM)	Minimal medium	Bacterial strain		
		Sm	Sm Sup. So^A^	Sm Sup. Lr^B^
Lactate	MM	1.78 ± 0.65 × 10^−2^	−5.14 ± 0.50 × 10^−2***^	−1.13 ± 1.08 × 10^−2**^
	MM+Glu	6.52 ± 0.91	3.84 ± 0.40**	2.95 ± 0.31**
	MM+Gly+Glu	5.00 ± 0.42	3.01 ± 0.49**	3.65 ± 0.33 × 10^−1***^
	MM+Lac+Glu	4.46 ± 0.41	−1.39 ± 0.25***	4.42 ± 0.19
	MM+Arg+Glu	6.03 ± 0.48	1.69 ± 0.43***	3.29 ± 0.26***
	MM+all	6.00 ± 0.29	−2.14 ± 0.35***	−0.46 ± 0.11***

For the culture of *S. mutans*, in order to enable it to produce lactate, the same concentration of glucose (5 mM) as in MM+Glu and MM+all was additionally added to MM+Gly, MM+Lac, MM+Arg. Glucose was also added to the probiotic supernatants of MM+Gly, MM+Lac, and MM+Arg. *S. mutans* was cultivated in minimal medium (MM), MM with glucose (MM+Glu), MM with glycerol and glucose (MM+Gly+Glu), MM with Lactic acid and glucose (MM+Lac+Glu), MM with arginine and glucose (MM+Arg+Glu) and all-full medium (MM+all). The lactate concentration of *S. mutans* cultivated in probiotic supernatants (test samples) and the lactate concentration in probiotic supernatants without *S. mutans* (control samples) were measured. The lactate production of *S. mutans* cultured in probiotic supernatants was calculated by subtracting the amount of lactate in control samples from the amount of lactate in corresponding test samples. Positive values indicate additional lactate production, negative values indicate uptake of lactate by bacteria. Dunnett’s test: *P < 0.05, **P < 0.01, ***P < 0.001, versus Sm.

### Inhibitory Effect of the Probiotic Supernatants on the Survival of *S. mutans*

**FIGURE 3:**
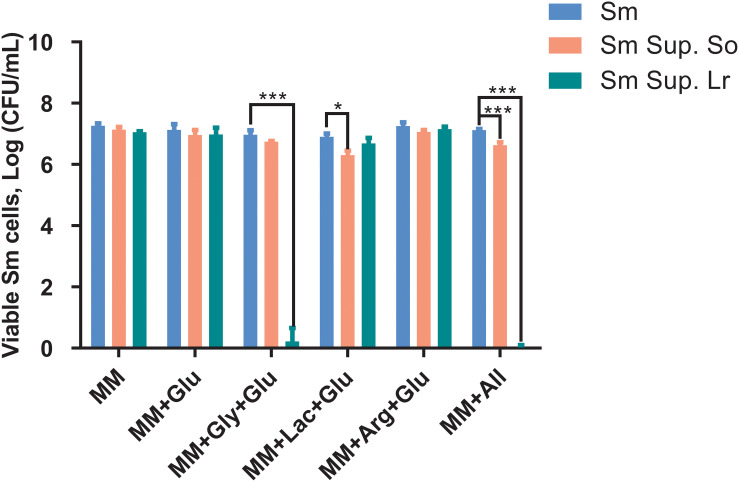
Changes in colony-forming units (Log CFU/mL, means ± standard deviations) of *S. mutans* after being cultured in the supernatant (Sup.) of *S. oligofermentans* (So) and *L. reuteri* (Lr). *S. mutans* was cultivated in minimal medium (MM), MM with glucose (MM+Glu), MM with glycerol and glucose (MM+Gly+Glu), MM with Lactic acid and glucose (MM+Lac+Glu), MM with arginine and glucose (MM+Arg+Glu) and all-full medium (MM+all) (controls), as well as supernatants of the probiotics. Glucose was additionally added to the MM+Gly, MM+Lac, and MM+Arg group of probiotic supernatants as it is described in section “Culture of *S. mutans* With Probiotic Supernatants.” Each experiment was repeated three times. Dunnett’s test: *P < 0.05, ***P < 0.001, versus controls.

When cultured in supernatants of *S. oligofermentans* and *L. reuteri*, survival of *S. mutans* was reduced compared with the controls (Figure 3). Specifically, treatment with the supernatant of *S. oligofermentans* resulted in a reduction in the viability of *S. mutans* in MM+Lac+Glu and MM+all by 0.6-log_10_ and 0.5-log_10_, respectively. Moreover, in MM+Gly+Glu and MM+all group, supernatant of *L. reuteri* yielded a reduction in viability of 6.1-log_10_ and 7.1-log_10_, respectively.

## Discussion

The present study investigated the potential of *S. oligofermentans* and *L. reuteri* to produce different antimicrobial substances under different environmental conditions, and tested the inhibitory effect of probiotic supernatants on lactate production and survival of *S. mutans.* We found that both probiotics produced H_2_O_2_ in different MM, and ammonia in MM+Arg. In MM containing glycerol, *L. reuteri* also produced reuterin. Both probiotics produced lactate when glucose was present. Cultivation in the supernatants of both probiotics reduced the metabolic activity and survival of *S. mutans* significantly and environment-specifically. It can be assumed that these effects were not associated with pH changes (which were minimal given the buffering capacity of the used media), but associated with the products generated by the probiotics (Castillo et al., 2000). We hence confirm our hypothesis.

The mechanism by which probiotics precisely interfere with cariogenic pathogens remains unknown. There are currently three possible explanations, including (1) release of bacteriocins (Martinez et al., 2013), (2) the antimicrobial effects through co-aggregation (Lang et al., 2010), (3) competition with cariogenic bacteria for nutrition and adhesion (Terai et al., 2015; Schwendicke et al., 2017). In this study, *S. mutans* was cultured in the supernatant of probiotics only. Hence, we can make certain assumptions as to how these supernatants interacted with *S. mutans*: In MM+Lac and MM+all, *S. oligofermentans* had produced large amounts of H_2_O_2_, which has likely impacted on the survival and lactate production of *S. mutans*. In MM+Gly and MM+all, *L. reuteri* produced large amounts of reuterin, with potentially even more pronounced effects on *S. mutans.* The production of ammonia by *S. oligofermentans* in arginine containing medium had only moderate inhibitory effects on *S. mutans* activity and survival. Note that we cannot fully exclude the observed effects to be associated with other, non-measured probiotic products present in the supernatant, but given the consistency and plausibility of the measured presence of H_2_O_2_ and reuterin and the observed effects on *S. mutans*, the outlined pathway of how probiotics impact on *S. mutans* seems likely.

H_2_O_2_ is the major antibacterial substance produced by *S. oligofermentans* (Zhang et al., 2010; Tong et al., 2020), as confirmed by our study. *S. oligofermentans* possesses three H_2_O_2_-forming enzyme: lactate oxidase (Lox), that catalyzes L-lactate and oxygen to produce H_2_O_2_ and pyruvate; pyruvate oxidase (Pox), that generates H_2_O_2_ by oxidizing pyruvate to acetate via acetyl coenzyme; L-amino acid oxidase, that catalyzes the production of H_2_O_2_ from amino acids and peptone (Tong et al., 2008; Liu L. et al., 2012). Our findings that H_2_O_2_ production was most pronounced in MM+Lac and MM+all, where lactic acid was available for Lox, suggest that Lox may play a role in H_2_O_2_ generation. The production of H_2_O_2_ in MM, MM+Glu, MM+Gly and MM+Arg was similar, presumably because *S. oligofermentans* had converted extracellular glucose into intracellular polysaccharides during precultivation in BHI+glucose. When cultivated in carbohydrate-limited MM variations, intracellular glucose or glycogen of *S. oligofermentans* was decomposed into pyruvate. Pyruvate can generate H_2_O_2_ and acetyl phosphate through Pox or can be converted into lactic acid by the lactate dehydrogenase. The additional availability of glucose in MM+all may further support Pox and Lox activity, as *S. oligofermentans* converted glucose into pyruvate and lactate, which act as substrates for Pox and Lox, respectively. Overall, *S. oligofermentans* requires a Pox-Lox synergy to produce the maximum amount of H_2_O_2_ (Liu L. et al., 2012).

Saliva and protein-rich foods contain abundant amounts of arginine. The arginine deiminase system degrades and metabolizes arginine to ammonia (Nascimento, 2018), which raises the pH (Liu Y. L. et al., 2012). For *S. oligofermentans*, we found that ammonia can be detected in MM+Arg and MM+all. The lower ammonia production in MM+all may be explained by excess glucose being present, with the arginine deiminase activity decreasing when glucose concentrations exceed 2 mM (Crow and Thomas, 1982; Kanapka and Kleinberg, 1983). Ammonia had only moderate effect on *S. mutans* activity and survival, which may be expected. Anti-caries effects of ammonia will be relevant nevertheless via altering the pH to a less cariogenic environment, hence supporting to rebalance remineralization over demineralization and preventing net mineral loss (Zaura and Twetman, 2019).

Reuterin is a multi-compound dynamic equilibrium system composed of 3-hydroxypropionaldehyde (3-HPA), its hydrate and dimer (Spinler et al., 2008) as well as acrolein. 3-HPA is a product of glycerol dehydration in the propanediol utilization (Pdu) pathway and is catalyzed by glycerol dehydratase (Chen and Hatti-Kaul, 2017). In reuterin solutions, acrolein and 3-HPA are interconverted, with acrolein being the active antimicrobial compound (Engels et al., 2016). In this study, we found *S. oligofermentans* to produce very little reuterin in MM+Gly and MM+all. *L. reuteri*, however, is known to possess the Pdu pathway to produce reuterin via fermentation of glycerol. After cultivation in the reuterin-containing supernatant of *L. reuteri*, *S. mutans* survival was reduced nearly completely. Our results are in line with those from clinical studies finding *L. reuteri* to be an efficacious probiotic to combat oral pathogens (Lin et al., 2017; Geraldo et al., 2019).

It has also been shown that *L. reuteri* is capable of producing H_2_O_2_ (Kang et al., 2011; Basu et al., 2019), and in our experiments, *L. reuteri* produced H_2_O_2_ in each MM variation. H_2_O_2_ is mainly produced by Pox (as described) and NADH oxidase (Nox) (Hertzberger et al., 2014), while it is unclear which enzymatic pathway was relevant in our setting. It was found that Pox synthesis was inhibited when glucose was abundantly available, while Nox was not essentially affected (Sedewitz et al., 1984). This was not the case in MM+Glu in our study. Hence, we assume that H_2_O_2_ was largely produced through NADH-dependent reactions, as shown for *Lactobacillus delbrueckii*, too (Marty-Teysset et al., 2000).

To assess the influence of the products generated by the probiotics on the lactate production and survival of *S. mutans*, *S. mutans* was cultivated in the probiotic supernatants. When cultivated in *S. oligofermentans* supernatant, lactate production by *S. mutans* in each MM variation group was significantly reduced. Since the supernatants of each MM variation media contained H_2_O_2_, the reason for this decrease in lactate production may be related to H_2_O_2_. Furthermore, ammonia produced in MM+Arg+Glu and MM+all may also neutralize lactic acid produced by *S. mutans*, reminding us that adjustment the alkali-generation potential of oral microbial may also have great potential. A similar reduction in lactate production was found when *S. mutans* was cultured in *L. reuteri* supernatants for all groups except MM+Lac+Glu. We consider that it is because the amount of H_2_O_2_ in MM+Lac+Glu was too small to reach an effective concentration.

Our findings agree with the study of Rossoni et al. (2018) in which they found the growth of *S. mutans* in planktonic cultures was inhibited by the bioactive substances released by *Lactobacillus* strains. In our study, almost no CFU were detectable in MM+Gly+Glu and MM+all of *L. reuteri* supernatant, proving reuterin as a potentially powerful antibiotic substance. *S. mutans* showed significantly lower survival in the culture of the supernatants in MM+Lac+Glu and MM+all of *S. oligofermentans*, which indicated that large amounts of H_2_O_2_ produced in a lactate-rich environment may have an inhibitory effect on the survival of *S. mutans*.

Overall, our study demonstrated that the products generated by the probiotics in the supernatants may inhibit the metabolic activity and survival of *S. mutans* and this effect was environment-specific. While it may well be that additional benefits emerge from the usage of viable probiotic, e.g., via co-aggregation (probably between *L. reuteri* and *S. mutans*), competition with cariogenic bacteria for nutrients and adhesive surfaces, and isolation of substrate or metal ions, our findings open up new therapeutic avenues. Using supernatant or specific isolated compounds for inhibiting cariogenic pathogens comes with the advantage of being safer, easier to dose, and any product having an extended shelf-life compared with living probiotics. Understanding the interactions between probiotics and cariogenic bacteria in simulated oral environments and identifying the underlying molecular mechanisms may support more effective and safe applications.

This study has several limitations. First, the complex oral conditions cannot be completely simulated *in vitro*. The impact of other bacteria species and the relevance of further proteins being available for bacterial metabolization will likely modify our findings. For the sake of interpretability, however, a simplified model such as ours seems useful. Second, our method for detecting specific bacterial substances produced by probiotics using colorimetric assays was not comprehensive; a more detailed metabolomic analysis may yield further insights. Similar, determining the CFU/mL of *S. mutans* in planktonic bacterial cultures to assess survival inhibition comes with limitations and does not fully reflect that probiotic effects in a clinical setting should target dental biofilms. However, both the colorimetric assay and the enumeration of planktonic bacteria via CFU/mL were chosen as they are easy to operate, reproducible and sufficient for the purposes of this study. Third, the culture time used in this study were optimized to capture, in a limited amount of time, the specific impact of the different metabolites. Different culture periods will be associated with different degrees of bacterial interaction. Last, there may be other mechanism, like end-product inhibition, as a possible non-specific mechanism that leads to a decrease in lactate production of *S. mutans*. However, the actual impact of this mechanism on our experimental results requires further verification.

In conclusion and within these limitations, the probiotic effects of *S. oligofermentans* and *L. reuteri* supernatants on the metabolic activity and survival of *S. mutans* were environment-specific through different pathways. Future studies as well as clinical applications should consider environment-specific probiotic actions on cariogenic pathogens.

## Data Availability Statement

All datasets generated for this study are included in the article/supplementary material.

## Author Contributions

HY performed the experiments, data analysis, and wrote the manuscript. PG and HY conceived and designed the study. PG guided the experiments and revised the manuscript. FS conceived the study, guided the design of the experiments, reviewed and edited the manuscript. All authors had approved the final version of the work.

## Conflict of Interest

The authors declare that the research was conducted in the absence of any commercial or financial relationships that could be construed as a potential conflict of interest.
